# Grossesse dans une corne rudimentaire: difficultés diagnostiques et prise en charge thérapeutique

**DOI:** 10.11604/pamj.2016.24.14.6659

**Published:** 2016-05-04

**Authors:** Nisrine Mamouni, Nabil Ghazal, Sanaa Erraghay, Chahrazed Bouchikhi, Abdelaziz Banani

**Affiliations:** 1 Service de Gynécologie-Obstétrique I, CHU Hassan II, Fès, Maroc

**Keywords:** Corne rudimentaire, diagnostic, traitement, pronostic, Rudimentary horn, diagnostic, treatment, prognosis

## Abstract

La survenue d'une grossesse dans une corne utérine rudimentaire est une situation obstétricale extrêmement rare et potentiellement grave, menaçant le pronostic materno-fœtal. Les auteurs rapportent cinq observations de grossesse dans une corne utérine rudimentaire, à travers lesquelles, ils relatent les difficultés sur le plan diagnostique ainsi que la prise en charge thérapeutique de cette entité pathologique, soulignant l'intérêt de l’échographie endovaginale, de l'IRM pelvienne et de la cœlioscopie dans le diagnostic précoce de ce type de malformation uterine.

## Abstract (en)

The occurrence of rudimentary horn pregnancy is an extremely rare and potentially serious obstetric entity, threatening maternal and fetal outcome. The authors report five cases of rudimentary horn pregnancy, the difficulties in making a proper diagnosis and the therapeutic management of this pathological entity, stressing the importance of transvaginal ultrasound, of pelvic MRI and laparoscopy in the early diagnosis of this type of uterine malformation.

## Introduction

Les utérus unicornes résultent d'un défaut de développement de l'un des deux canaux de Müller. Ils sont fréquemment associés à une corne rudimentaire lorsqu'un des deux canaux ne se développe que partiellement [1]. Cette corne peut être pleine ou au contraire le siège d'une cavité, et c'est dans ce dernier cas qu'elle peut communiquer avec l'utérus [1]. Les utérus unicornes avec corne rudimentaire sont responsables de plusieurs complications dont les plus graves sont les anomalies de la placentation et les grossesses ectopiques [2, 3]. L'incidence des grossesses sur corne rudimentaire est estimée à 1/100 000 à 1/140 000 [4]. Embryologiquement, l'utérus bicorne avec corne rudimentaire correspond à un défaut de progression d'un des deux canaux de Müller entre la sixième et la neuvième semaine de gestation. Cette corne rudimentaire peut contenir ou non une cavité avec endomètre pouvant alors être le siège d'implantation d'une grossesse.

## Patient et observation

**CAS 1**: madame MK, âgée de 28 ans, sans antécédents pathologiques notables, primigeste, qui s'est présentée aux urgences pour des douleurs pelviennes aiguës associées à des métrorragies de faible abondance faite de sang rouge sur une grossesse de 13 SA, et chez qui l'examen clinique trouve une TA à 75/45 mmHg, une défense abdominale généralisée, un utérus faisant 12 SA mou et un cri de Douglas. La patiente a bénéficié d'une échographie obstétricale objectivant une grossesse mono fœtale non évolutive avec un épanchement intra péritonéal de grande abondance. Une laparotomie en urgence a été réalisée. A l'exploration: un hémopéritoine aspiré d'environ 2 litres. La grossesse s'est développée dans une corne utérine rudimentaire droite rompue. On a réalisé une exérèse chirurgicale de la corne rompue et de la trompe homolaterale. Les suites opératoires ont été simples.

**CAS 2**: madame JG, âgée de 30 ans, cinquième geste, quatrième pare, admise pour prise en charge des douleur pelviennes aigues sur grossesse 18SA et chez qui l'examen clinique trouve une TA 9/6mmhg, sensibilité abdominale généralisée. L’échographie obstétricale en faveur d'une grossesse monofœtale non évolutive 18SA avec un épanchement intra péritonéal de grande abondance. La patiente admise au bloc opératoire pour laparotomie en urgence. L'exploration per-opératoire a trouvé une grossesse sur corne rudimentaire rompue avec expulsion du fœtus et du placenta en intra abdominal. Une résection de la corne rudimentaire avec salpingectomie homolatérale a été réalisée et les suites post-opératoires ont été simples.

**CAS 3**: madame FD, âgée de 39 ans, deuxième geste, primipare, porteuse d'un utérus cicatriciel admise pour prise en charge des douleurs abdominopelviennes sur grossesse 14SA. L'examen trouve patiente stable sur plan hémodynamique avec un abdomen distendu et un cri de douglas. Culdocentése positive patiente admise au bloc opératoire pour laparotomie en urgence. L'exploration per-opératoire a trouvé une corne rudimentaire rompue avec expulsion du fœtus et du placenta en intra abdominal. Une résection de la corne rudimentaire avec salpingectomie homolatérale a été réalisée et les suites post-opératoires ont été simples.

**Figure 1:**
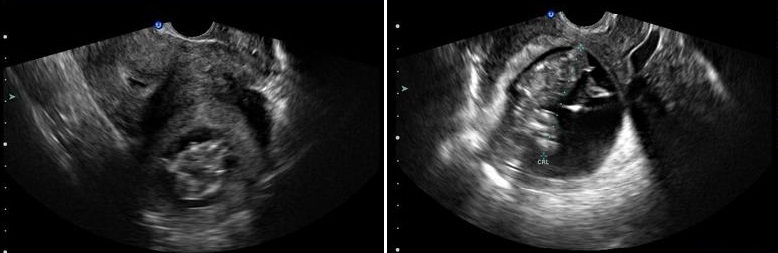
Echographie pelvienne: cavité uterine vide avec presence en laterouterin d'un sac gestationnel embyonné entouré d'une couronne de tissu myometrial

**Figure 2:**
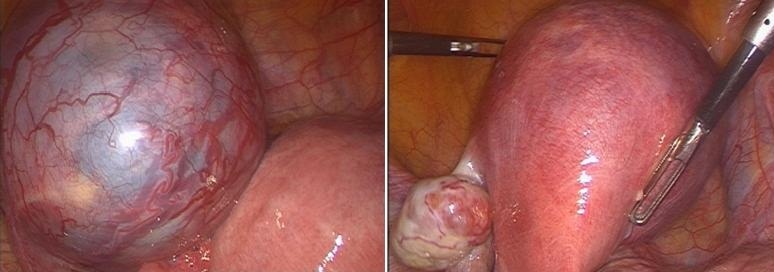
Exploration cœlioscopique montrant la présence d'une corne utérine rudimentaire

**Figure 3:**
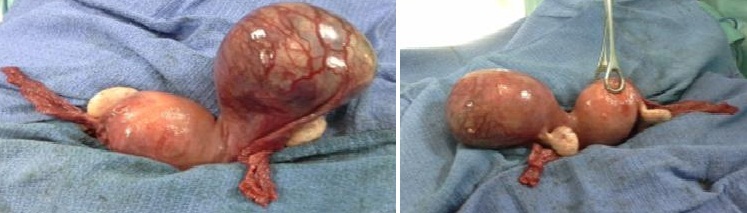
Exploration per opératoire: corne rudimentaire gauche gravide attachée par un collet à l'hemimatrice droite

**CAS 4**: madame HK, âgée de 33 ans, sans ATCD pathologique notable, troisième geste, deuxième pare, admise pour des algies pelviennes avec métrorragies de sang noirâtre de faible abondance sur une aménorrhée de 3 mois. L'examen clinique trouve une patiente stable sur le plan hémodynamique, HU à 2 TDD de la symphyse pubienne. Col d'aspect normal et absence de saignement, au toucher vaginal, le co lest fermé et la taille de l'utérus difficile à apprécier vu le pannicule adipeux. L'Echo pelvienne a objectivé la présence de deux matrices utérines (Figure 1), la première de taille normale ligne d'interface visualisée en totalité endomètre épaissi, La 2^eme^ contenant un sac gestationnel avec embryon de 12 SA, absence d’épanchement intra péritonéal. Une cœlioscopie diagnostique a objective la présence d'un utérus bicorne avec une corne utérine gauche rudimentaire augmentée de taille communicant avec la première au niveau de la région isthmique (Figure 2). Après conversion en laparotomie une résection de la corne rudimentaire a été réalisée ainsi qu'une salpingectomie homolaterale. Les suites opératoires ont été simples (Figure 3).

**Figure 4:**
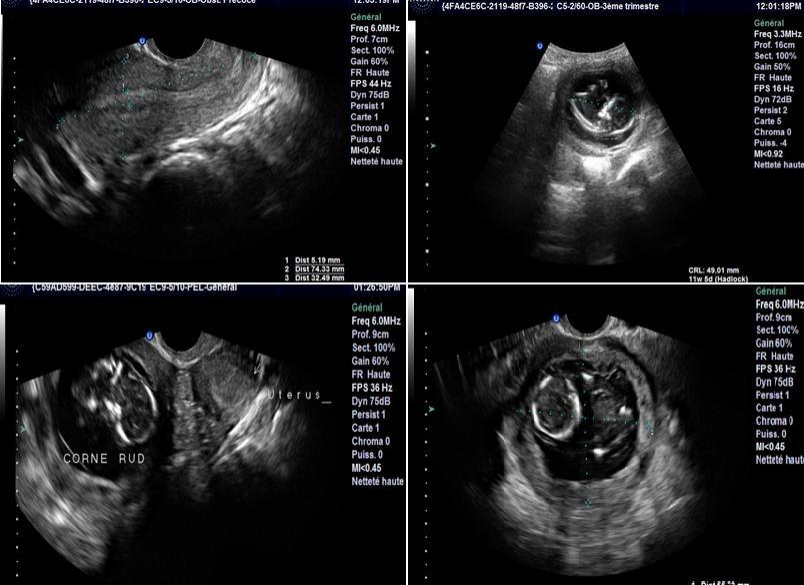
Echographie pelvienne: la présence du sac gestationnel entouré du tissu myometrial fin au niveau de la corne rudimentaire

**Figure 5:**
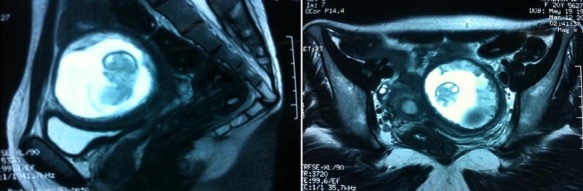
IRM pelvienne: coupes axiale et sagittale: utérus bicorne unicervical avec hémimatrice gauche borgne rudimentaire

**CAS 5**: madame JH, âgée de 20ans, nulligeste, sans ATCD patho notables, admise pour algie latéro pelvienne gauche aiguës isolées sur aménorrhée de 3 mois. L'examen Clinique trouve une sensibilité pelvienne avec un utérus faisant 12 SA. Echo pelvienne objective la présence d’ utérus de contours réguliers faisant 64/29 mm avec un endomètre épaissi, avec présence en sus et latéro utérine droit d'une image faite d'un sac gestationnel avec embryon faisant 11 SA sans activité cardiaque, le tout entouré par une structure myométriale (Figure 4). Une IRM pelvienne réalisée a été en faveur d'un utérus bicorne uni cervical avec hémimatrice gauche borgne rudimentaire (Figure 5). Une intervention par cœlioscopie a été réalisée avec l'introduction du trocard n°10 en periombilical, les deux trocads n°5 ont été introduits a travers deux incisions aux niveaux des deux fosses iliaques. L'exploration a objectivé la présence d'un utérus bicorne avec une corne rudimentaire gauche augmenté de taille, les annexes ont été sans particularités. Pour des raisons techniques, le geste chirurgical a été converti en laparotomie avec la réalisation d'une incision type Pfannentiel. Avec ligature section du ligament rond du coté gauche puis ligature section du ligament tubo -ovarien gauche et du ligament utéro ovarien gauche. La résection de la corne rudimentaire contenant la grossesse arrêtée a été réalisée avec salpingectomie homolaterale. A l'ouverture de la pièce, présence d'un sac gestationnel contenant un embryon momifié de 12 SA.

## Discussion

Les malformations utérines affectent 0,5% des femmes. Cinq pour cent d'entre elles sont des utérus unicornes [5]. L'incidence des grossesses sur corne rudimentaire est estimée à 1/100 000 à 1/140 000 [5]. Dix pour cent de ces cornes rudimentaires communiquent avec la corne utérine principale et 35% ont une cavité [6]. Cette cavité comporte rarement un endomètre fonctionnel. On retrouve une légère prédominance de cette corne rudimentaire du côté droit probablement due au fait que le canal de Müller gauche progresse plus caudalement que le droit [6]. Le développement des canaux de Müller et des canaux de Wolff étant étroitement lié, on retrouve dans 38% des cas une malformation de l'arbre urinaire associée, souvent une agénésie rénale unilatéral mais parfois aussi un rein pelvien ou un rein en fer à cheval [7].

L'endomètre qui la tapisse est parfois fonctionnel, exposant alors au risque d'anomalie de la placentation [1, 2]. La survenue d'une grossesse dans cette corne rudimentaire résulterait de la migration intra péritonéale des spermatozoïdes ou de l'ovocyte fécondé. Lorsque cette malformation est méconnue, la localisation de la grossesse est difficile en anténatal comme en témoigne notre observation avec un diagnostic erroné de fibrome prævia qui correspondait en réalité à l'hémi-utérus normal. Il est important d'insister sur l'importance de la première échographie obstétricale dont un des buts est de faire le diagnostic de grossesse intra-utérine et de vérifier l'absence de malformation utérine.

Le diagnostic peut être fait par l’échographie, surtout endovaginale, par l'hystérosalpingographie et parfois par la tomodensitométrie ou l'imagerie par résonance magnétique. Il est difficile de faire le diagnostic précoce d'une grossesse sur corne rudimentaire. L’échographie endovaginale paraît être un bon moyen diagnostique de ces grossesses surtout au premier trimestre. Ce diagnostic pourra être étayé par une imagerie par résonance magnétique. Lorsqu'un utérus bicorne avec corne rudimentaire est diagnostiqué en dehors de toute grossesse, il est conseillé de réaliser une hémi hystérectomie. Il a été décrit des interventions par laparoscopie y compris sur corne gravide [8, 9]. Lors du premier trimestre, la grossesse sur corne rudimentaire peut être différenciée d'une grossesse tubaire ou abdominale par la présence de tissu myométrial entourant le sac gestationnel et la présence d'un placenta bien individualisé. De plus, elle peut être suspectée par l'absence de continuité entre le col et la poche des eaux (échographie endovaginale) et également par la présence d'un utérus bicorne avec asymétrie entre les deux cornes. Il est néanmoins difficile de faire le diagnostic de cette malformation utérine avant la laparotomie effectuée en urgence pour état de choc hémorragique [10]. Il est fait dans moins de 5% des cas avant laparotomie [8]. Il est alors conseillé de réaliser l'exérèse de la corne rudimentaire ainsi que de la trompe homolatérale afin de prévenir une grossesse ectopique ultérieure si la trompe controlatérale paraît fonctionnelle. Ces grossesses sont également à risque de placenta acreta et percreta, probablement du fait de la mauvaise qualité de l'endomètre et de sa faible décidualisation [10, 11].

Ces grossesses évoluent très majoritairement (90%) vers la rupture de la corne rudimentaire, le plus souvent au deuxième trimestre de la grossesse dans un tableau d'inondation péritonéale avec un taux de sauvetage fœtal faible, de l'ordre de 2% [2, 6]. Rarement, l'extensibilité de la corne rudimentaire permet d'approcher le terme et d'extraire un enfant vivant comme dans notre observation. Il a également été décrit des cas de grossesses gémellaires dont une était dans la corne rudimentaire et l'autre dans la corne fonctionnelle, ce qui représenterait 5,3% des grossesses d'après Nahum [5].

Le traitement doit rester le même quelle que soit la malformation utérine, c'est-à- dire l'ablation de la corne utérine rudimentaire et de la trompe homolatérale. Certains auteurs ont même propose de réaliser cette intervention lors de la découverte d'un utérus pseudounicorne avec corne rudimentaire en dehors de toute grossesse, sans attendre un accident obstétrical [8, 9]. La voie d'abord cœlioscopie est en 1^ere^ intention (trocart ombilical et 3 trocarts sus pubien) hors grossesse et pendant grossesse.

Environ trios cas de grossesse sur corne rudimentaire découverte au deuxième trimestre ont été traitée par voie laparoscopique. L'extraction de la pièce opératoire peut se faire par élargissement de l'incision, l'extraction à travers une colpotomie postérieure ou par morcellation après injection fœtale de chlorure de potassium [12]. Les futures grossesses nécessiteront alors une surveillance extrêmement rapprochée, la patiente devant être informée et consciente des risques encourus. Les obstétriciens prenant en charge ces patientes doivent être conscients du risque sérieux de rupture utérine au cours de la grossesse. Une césarienne avant tout travail à terme sera fortement recommandée.

## Conclusion

La grossesse dans une corne utérine rudimentaire constitue une forme rare de grossesse ectopique, de diagnostic difficile, pouvant être révélé sur un mode aigu secondairement à une rupture utérine, mettant alors en jeu le pronostic vital fœtal et maternel. Sa prise en charge met habituellement en jeu l'excision de la corne rudimentaire. Pour éviter une récidive plus précoce et plus sévère.
